# HIV prevention and care services for female sex workers: efficacy of a targeted community-based intervention in Burkina Faso

**DOI:** 10.7448/IAS.18.1.20088

**Published:** 2015-09-14

**Authors:** Isidore T Traore, Nicolas Meda, Noelie M Hema, Djeneba Ouedraogo, Felicien Some, Roselyne Some, Josiane Niessougou, Anselme Sanon, Issouf Konate, Philippe Van De Perre, Philippe Mayaud, Nicolas Nagot

**Affiliations:** 1 Department of Clinical Research, Centre Muraz, Bobo-Dioulasso, Burkina Faso, West Africa; 2 Department of Public Health, Centre Muraz, Bobo-Dioulasso, Burkina Faso, West Africa; 3 Centre of International Research for Health, University of Ouagadougou, Ouagadougou, Burkina Faso, West Africa; 4 UMR 1058, INSERM/University of Montpellier/EFS, Montpellier, France; 5 CHRU Montpellier, Montpellier, France; 6 Department of Clinical Research, London School of Hygiene & Tropical Medicine, London, UK

**Keywords:** female sex workers, HIV, incidence, Africa

## Abstract

**Introduction:**

Although interventions to control HIV among high-risk groups such as female sex workers (FSW) are highly recommended in Africa, the contents and efficacy of these interventions are unclear. We therefore designed a comprehensive dedicated intervention targeting young FSW and assessed its impact on HIV incidence in Burkina Faso.

**Methods:**

Between September 2009 and September 2011 we conducted a prospective, interventional cohort study of FSW aged 18 to 25 years in Ouagadougou, with quarterly follow-up for a maximum of 21 months. The intervention combined prevention and care within the same setting, consisting of peer-led education sessions, psychological support, sexually transmitted infections and HIV care, general routine health care and reproductive health services. At each visit, behavioural characteristics were collected and HIV, HSV-2 and pregnancy were tested. We compared the cohort HIV incidence with a modelled expected incidence in the study population in the absence of intervention, using data collected at the same time from FSW clients.

**Results:**

The 321 HIV-uninfected FSW enrolled in the cohort completed 409 person-years of follow-up. No participant seroconverted for HIV during the study (0/409 person-years), whereas the expected modelled number of HIV infections were 5.05/409 person-years (95% CI, 5.01–5.08) or 1.23 infections per 100 person-years (*p*=0.005). This null incidence was related to a reduction in the number of regular partners and regular clients, and by an increase in consistent condom use with casual clients (adjusted odds ratio (aOR)=2.19; 95% CI, 1.16–4.14, *p*=0.01) and with regular clients (aOR=2.18; 95% CI, 1.26–3.76, *p*=0.005).

**Conclusions:**

Combining peer-based prevention and care within the same setting markedly reduced the HIV incidence among young FSW in Burkina Faso, through reduced risky behaviours.

## Introduction

In West Africa, female sex workers (FSW) remain the main core group involved in HIV transmission dynamics [1, 2]. More than 75% of HIV infections acquired by heterosexual West African men are attributed to sexual intercourse with FSW [3]. In this region, almost half of FSW clients are bridging populations reporting unprotected sexual intercourse with both FSW and other women from the general population, mainly their wives or stable partners [4]. In Burkina Faso, the HIV prevalence [5, 6] was 1.2% in 2010 in the general population, but 13-fold higher among FSW (16%) [7]. Many situational analyses reported a high proportion of native part-time FSW [8, 9] who are highly stigmatized due to social discrimination, criminalization of the street-based prostitution and police repression. In 2009, in Ouagadougou, more than 65% of HIV-prevention interventions were discontinued when funding ceased (Berthe, 2009) [10]. This environment, combined with poverty, is directly responsible for their weak power in negotiating condom use [9], low access to sexual and reproductive health (SRH) care services [11] and low exposure to safer sex education.

In order to reduce the spread of HIV in generalized and concentrated epidemic settings, the implementation of interventions increasing FSW access to both HIV prevention and care services is crucial [6, 12]. However, the design and implementation of these interventions are complex mainly due to the difficulty in reaching the target population such as part-time sex workers, who may not acknowledge being “sex workers,” but who play an increasing role in commercial sex, with a similar HIV risk as full-time FSW [2, 8, 9, 13].

Because secondary prevention of HIV infection through treatment has become crucial to impact the HIV transmission dynamics [14–16], dedicated services with tailored support for antiretroviral therapy (ART) adherence are likely to improve access to care, therapeutic success and prevention of secondary transmission of HIV, including with resistant viruses [17, 18].

Our group showed that such an approach, with a strong community involvement, helped in achieving high rates of virological and immunological success of ART [17] with a major impact on infectiousness for sexual transmission [15], but, so far, no data are available on the impact of such a combined intervention on HIV incidence, particularly among young FSW who have recently started commercial sex. The evaluation of such combined intervention is complex. A consensus emerged for not using a randomized controlled trial because using a control group without any intervention would not be ethically acceptable in this vulnerable population [19–21]. The present study aimed at estimating the impact of this combined approach targeting young FSW in Burkina Faso, focusing on HIV incidence and unsafe sex practices. The evaluation of the intervention relied both on mathematical modelling, a viable alternative to randomized controlled trials [22–25] and on an estimation of baseline incidence using baseline HIV prevalence [26, 27].

## Methods

### Study design

From 2009 to 2011, we conducted a prospective, interventional cohort among HIV-uninfected FSW in Ouagadougou, with quarterly follow-up for a maximum of 21 months. The HIV incidence under intervention was compared with a modelled HIV incidence in the cohort in the absence of intervention, using data collected at the same time from another survey among clients of sex workers in the same city [7].

### Study population

Women who declared receiving money or goods in exchange for sexual services [20, 21], were born in Burkina Faso, were aged between 18 and 25 years, had at least three sexual contacts per week and three different sexual partners during the last three months were eligible for this cohort. Non-inclusion criteria included a positive urinary pregnancy test, or a plan to move out of Ouagadougou in the next two years. For this analysis, only women uninfected at baseline were considered.

In Burkina Faso, sex workers include full-time FSW (street-based) for whom sex work is the main activity, and part-time FSW who have occasional clients (bar workers, fruit sellers, etc.) without considering themselves as FSW [9]. Actual involvement in commercial sex was visually ascertained at night by study peers at the work place.

### Study intervention

First, we carried out a formative research to address structural risk factors, through in-depth interviews and focus-group discussion conducted with key stakeholders (FSW, bar managers, NGOs and national institutions working in the field of HIV/AIDS, hygiene and security). This phase was useful to adapt the intervention package to the SRH and HIV needs of the FSW, and to get the support of stakeholders. A community advisory board composed of local AIDS, NGOs and FSWs was established and worked closely with the study team (Berthe, 2009) [10].

The intervention consisted of peer-led education sessions, free provision of STI syndromic management, condoms and hormonal contraceptives, psychological support and free general medical and HIV care (for those HIV infected at screening or seroconverting within the study period) [17, 18]. Peer-led education sessions were conducted every day at the study clinic and weekly in the sex work venues, addressing seven themes including HIV testing, STI diagnosis and treatment, genital herpes, condom use, condom negotiation, family planning and drug adherence.

At each follow-up visit, STI symptoms were assessed by systematic gynaecological examination and treated by syndromic management according to national guidelines.

### Procedures and follow-up

During the formative research, sex work venues were geo-mapped using geographic information system. Potentially eligible FSW were contacted by peers at their workplace and invited to attend a screening visit. Eligible women were invited one week later for enrolment after full information and written informed consent.

At enrolment and subsequent visits every three months for a minimum of 12 months, trained social workers administered a standardized questionnaire documenting sexual behaviours and alcohol consumption during the previous week, including the number and type of sexual partners: paying clients (casual clients), regular partners (living under the same roof as a couple or being in love) and regular clients (paying or non-paying sex partners different from regular partners and casual clients). After physical examination by the study physician, urine, vaginal and endocervical samples were collected, as well as a blood sample after a voluntary counselling session for HIV.

Contact tracing was organized by peers at home or worksite according to participant preference. To preserve confidentiality, a unique study number was assigned to each participant and used for all study documents. Participant files were stored in secure filing cabinets. The National Health Research Ethics Committee of Burkina Faso and the research ethics committee of the London School of Hygiene & Tropical Medicine (UK) approved the study protocol.

### Laboratory procedures

HIV infection was detected by rapid tests using Determine (Laboratoires Abbot, Japan) and Genie II HIV-1/HIV-2 (Bio-Rad, Marnes la Coquette, France) [28]. Among those having indeterminate results, samples were tested using HIV-1 RNA PCR (Biocentric, Bandol, France). Serum samples were tested by a type-specific IgG ELISA for herpes simplex virus type-2 (HSV-2; KALON HSV-2 IgG, Kalon Biological Ltd, Guildford, UK). Vaginal wet mounts were prepared at the study clinic for detection of “clue cells,” motile *Trichomonas vaginalis* and yeast cells. Vaginal swabs from the lateral vaginal walls were gram stained and examined for bacterial vaginosis using the Nugent's scoring method (score≥7) [29] and also for *Candida albicans*. Pregnancy tests (Vikia HCG-S) were done at the study clinic on urine samples.

### Study outcomes

The primary outcome was HIV incidence. Secondary outcomes included changes in mean number of sexual partners during follow-up and condom use with these sexual partners at the last sexual intercourse. Consistent condom use was defined by a systematic use of condoms during the last week for casual clients and during the last month for regular partners and regular clients.

### Statistical analyses

#### Estimation of the expected HIV incidence during the follow-up

**Figure 1:**
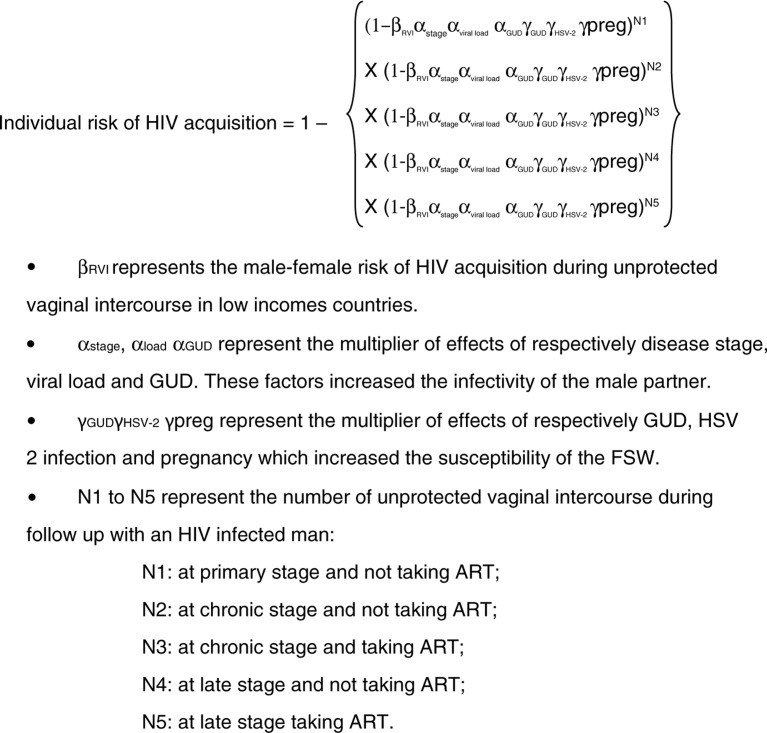
Transmission model to estimate the expected HIV incidence in the absence of intervention.

In order to estimate the HIV incidence in the absence of intervention, we used a transmission model parameterized with factors related to FSW sexual partners (HIV infection, HIV disease stage, ART), to the FSW themselves [HSV-2 infection, genital ulcer disease (GUD)] and to risk of male-to-female HIV transmission (0.38%; 95% CI, 0.13–1.1%) as reported in a recent meta-analysis [30]. A Bernoulli mathematical model with weighted risk factors was used to estimate the individual relative risk of HIV acquisition during follow-up [26] (Figure 1).

Because HIV viral load of FSW sexual partners was not available, we made the assumption that 80% of those receiving ART had an undetectable HIV viral load.

#### Number of unprotected sexual acts during follow-up, according to HIV, ART and disease-stage status of FSW sexual partners (N1–N5)

We multiplied the number of sexual intercourses with casual clients during the previous week (as reported by FSW at baseline), by the total number of person-weeks and by the rate of non-condom use with casual clients (estimated at screening visit). Then, we obtained the total number of unprotected sexual intercourses with casual clients without the intervention during follow-up. The number of unprotected sexual intercourses with both regular clients and regular partners were also calculated alike. The total number of all unprotected sexual intercourses during follow-up without intervention was obtained by summing the total numbers of unprotected sexual intercourses with casual clients, regular clients, and regular partners.

To obtain the total number of unprotected sexual acts with each group of partners (N1–5), we used the reported HIV prevalence in Ouagadougou among these sexual partners (3.2%; 95% CI, 1.3–5.5) [7], the proportion of HIV-infected men on ART in Burkina Faso (19.2%; 95% CI, 17.6–27.5) [5] and a conservative assumption that 95% of HIV-infected men are at the chronic stage, 2.5% are at the primary stage and 2.5% at the late stage. These latter two stages have the highest score of HIV transmission risk compared with the chronic stage (4.98; 95% CI, 2.0–12.39 and 3.49; 95% CI, 1.76–6.92, respectively) [26].

#### Estimation of the impact of the intervention

To estimate the expected number of HIV infections without intervention during the same follow-up time, we included in the Bernoulli-weighted model the average rates of the per-act male-to-female risk, the HIV prevalence among male partners and the scores related to factors increasing infectivity of sexual partners and susceptibility of FSW [26]. The bootstrap mean and 95% CI of the individual relative risk of HIV acquisition was multiplied by the total amount of follow-up in years.

Finally, the expected number of HIV infections was compared with the observed number of HIV infections using a Poisson distribution.

We also carried out sensitivity analyses using the lower and upper bounds of the 95% CI of the factors included in the Bernoulli model. For each scenario, observed and expected number of HIV infections were also compared using a Poisson distribution.

#### Model validation

In the absence of incidence data in a control group, we also used HIV prevalence at screening among young FSW who participated in sex work for less than one year, to estimate HIV incidence in the absence of the intervention [31, 32]. This approach, which assumes that women are not HIV-infected before sex work, is recommended by UNAIDS [31].

#### High-risk behaviours overtime

The number of sexual partners was categorized as above or below the third quartile value of the number of casual clients at baseline, and as none or any for regular clients and regular partners.

The trend over time of key determinants of sexual behaviours (number and types of sexual partners and condom use rate) were described using a random effect-logistic approach [33].

Because of high uncertainty in the measurement of behavioural data, all variables with a *p*<0.3 in univariable analysis were included in the multivariable models not to miss any important factor of interest [34]. In multivariable models, FSW category was kept in all final models and we used backward elimination to identify other covariates to include in the final models. Those not statistically significant at 5% significance level were withdrawn from the models. Complete case analysis was used to handle missing data. All analyses were conducted using SAS version 9.2.

## Results

### Participants’ characteristics

**Figure 2:**
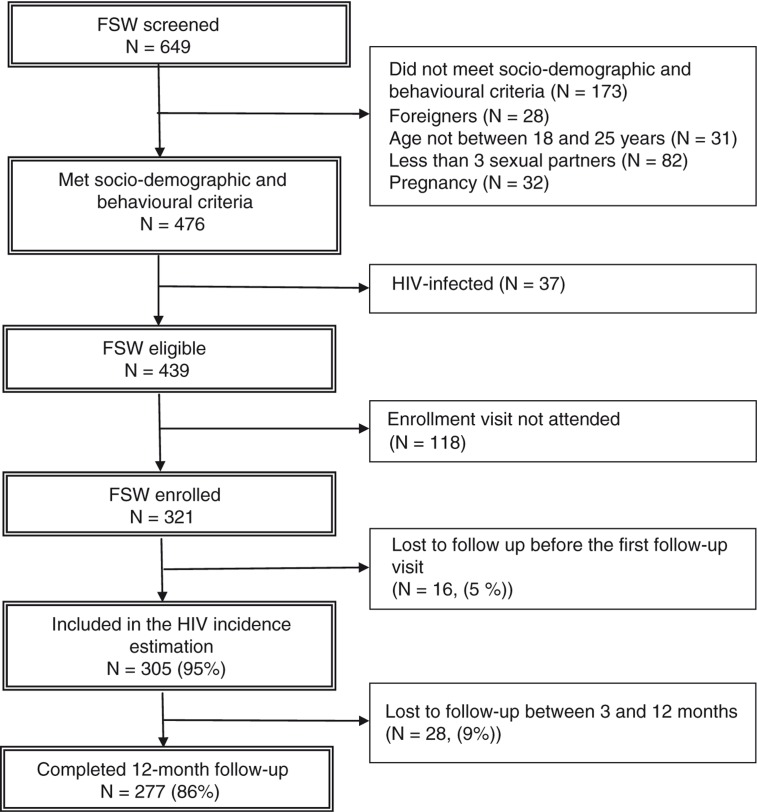
Flow chart of participants from screening to the 12-month follow-up visit in Ouagadougou.

Among the 476 FSW screened, the HIV prevalence rate was 7.8% (95% CI, 5.7–10.5). We enrolled 321 FSW in the cohort (Figure 2).

The FSW enrolled were more likely full-time FSW with high number of clients and previous pregnancies compared to eligible FSW not enrolled. The latter were also more likely to have an earlier age of sex work debut (Table 1).

**Table 1:** Baseline participant characteristics and comparison with potentially eligible female sex workers who missed their enrolment visit in Ouagadougou

	Enrolled *N*=321	Eligible but not enrolled *N*=118	
		
	*n* (%) or median [IQR]	*N* (%) or median [IQR]	*p*
Socio-demographic characteristics			
Age (years)	21 [19–23]	20 [19–23]	0.42
Marital status (married or cohabiting)	109 (34)	31 (26)	0.13
Sex work status			<0.001
Professional	121 (38)	23 (20)	
Non-professional	200 (62)	94 (80)	
Education			0.29
None	87 (27)	32 (27)	
1–6 years (primary)	145 (45)	45 (38)	
≥7 years (secondary or superior)	89 (28)	41 (35)	
Median monthly income (€)	91 [53–149]	63 [38–114]	<0.001
Sex work is the main income source	211 (66)	60 (51)	0.003
Drug and alcohol consumption			
Drug^a^ consumption	14 (4)	–	–
Any alcohol consumption	202 (63)	68 (58)	0.37
Previous HIV-testing	231 (72)	74 (63)	0.06
Child desire	67 (21)	25 (21)	1.00
Sexual behaviours			
Age of sex debut (years)	16 [15–18]	17 [15–18]	0.04
Duration of sex work (years)	1 [0.1–3.0]	1 [0.0–2.0]	<0.001
Number of casual clients (previous week)	2 [1–3]	1 [1–1]	0.01
>1 regular clients (last month)	136 (42)	27 (23)	<0.001
>1 regular partner (last month)	59 (19)	21 (18)	0.88
>1 previous pregnancy	93 (29)	22 (19)	0.03
Always condom use with casual clients	273 (95)	86 (93)	0.45
Always condom use with regular clients	150 (77)	31 (70)	0.43
Always condom use with regular partner	128 (50)	59 (56)	0.29
Vaginal infections			
Vaginal candidiasis	36 (13)	15 (16)	0.38
Bacterial vaginosis	37 (13)	12 (13)	0.73
STI			
*Trichomoniasis vaginalis*	8 (3)	3 (4)	1.00
HSV-2 infection	91 (28)	–	–

a Include cannabis, volatile solvents, hallucinogens and amphetamines.

At enrolment, the median age of participants was 21 years [interquartile range (IQR) 19–23], the median number of clients the week before the enrolment visit was 2 (IQR, 1–3) and the median numbers of regular clients and regular partners during the month prior to enrolment were 1 (IQR, 0–2) and 1 (IQR, 1–1), respectively. Overall, 28% of FSW were HSV-2 seropositive, whereas 3% had *Trichomonas vaginalis* infection (Table 1).

### Observed and expected HIV incidence

Among 305 FSW who completed at least one follow-up visit and who were included in the incidence analysis, the median follow-up time was 16.8 (IQR, 13.6–18.9) months (Figure 2).

No participant seroconverted during the 409 person-years of follow-up, whereas 5.05 (95% CI, 5.01–5.08) HIV infections were expected without intervention (*p*=0.005), that is, 1.23 infection per 100 person-years.

In the sensitivity analyses, the expected number of HIV infections in the absence of the intervention ranged between 0.14 and 42.51 (*p*=0.86 and *p*<0.001 when comparing with 0 infection, respectively). The intervention showed a significant protective impact on HIV incidence in all but the unlikely situation where the values of parameters would stand at the lowest bound of the 95% confidence interval of their estimation (Table 2).

**Table 2:** Model parameters and sensitivity analyses for the expected number of HIV infections in the absence of the intervention among female sex workers in Ouagadougou

	Expected number of HIV infections during follow-up		
	
	Lower limit of the 95% CI	Median limit of the 95% CI	Upper limit of the 95% CI
Parameters from the literature			
HIV prevalence among clients of FSW [35]	0.0134	0.032	0.0546
Per-act transmission risk [30]	0.0013	0.0038	0.011
Score GUD [26]	1.3	2.58	5.69
Score HSV-2 infection [26]	1.7	3.1	5.6
Score HIV stage [26]			
Chronic stage	1		
Primary stage	2.00	4.98	12.39
Late stage	1.76	3.49	6.92
Score for detectable viral load [26]			
Undetectable	0.06	0.14	0.34
Detectable	0.51	1.72	5.75
Estimations			
Number of unprotected vaginal sexual acts during follow up with HIV-positive men at:			
Primary stage and not taking ART (N1)	4	10	16
Chronic stage and not taking ART (N 2)	126	295	453
Chronic stage and taking ART (N3)	27	70	171
Late stage and not taking ART (N4)	3	8	12
Late stage and taking ART (N5)	1	2	6
Number of expected infections during follow-up among FSW *n* (95% CI)	0.14 (0.14–0.15)	5.05 (5.01–5.08)	42.51 (42.28–42.74)
*p*^a^	0.86	0.005	<0.001

a When compared with the 0 observed HIV infection the follow-up.

#### Model validation

The estimated HIV incidence deriving from the baseline prevalence among recent FSW was 20/409 person-years, that is, 4.9/100 person-years (95% CI: 3.2–7.4). This was consistent with a significant impact of the intervention package (*p*<0.001). It also suggests that the final model (using the median limit of the CI 95% of parameters) was likely conservative and therefore tended to underestimate the effect of the intervention.

### Changes in sexual behaviours

#### Casual clients

Although the average number of casual clients did not change during follow-up, the odds of consistent condom use significantly increased [adjusted odds ratio (aOR)=2.19; 95% CI, 1.16–4.14]. This odds was significantly reduced among women who had previous pregnancies (aOR=0.74; 95% CI, 0.57–0.95). Full-time FSW used condom more systematically with casual clients than part-time FSW (Table 3).

**Table 3:** Predictors of consistent condom use with casual clients during follow-up of female sex workers in Ouagadougou

	Univariable model		Final multivariable model^b^	
		
Characteristics	Odds ratio (95% CI)	*p*	Adjusted odds (95% CI)	*p*
Time (3 months)	2.04 (1.09–3.81)	0.02	2.19 (1.16–4.14)	0.01
Age ≥22 years^a^	0.83 (0.46–1.52)	0.55		
Married or cohabiting	0.52 (0.27–1.00)	0.05		
Professional sex workers	1.98 (0.91–4.30)	0.08	2.47 (1.12–5.45)	0.02
Education^a^		0.53		
None	1			
1–6 years (primary school)	1.40 (0.70–2.82)			
≥7 years (≥secondary school)	1.51 (0.68–3.36)			
Age of sex debut (years)^a^	1.00 (0.88–1.14)	0.95		
Duration of sex work (years)^a^	1.12 (0.96–1.31)	0.13		
Number of clients (previous week)	1.06 (0.97–1.16)	0.22		
Number of regular partners (last month)	0.78 (0.46–1.32)	0.36		
Number of previous pregnancies^a^	0.78 (0.61–1.00)	0.05	0.74 (0.57–0.95)	0.01
Hormonal contraception	1.35 (0.66–2.77)	0.41		
Previous HIV testing^a^	0.81 (0.40–1.60)	0.53		
Any alcohol consumption^a^	0.46 (0.23–0.94)	0.03		
*Vaginal candidiasis*	0.74 (0.26–2.16)	0.58		
*Bacterial vaginosis*	0.66 (1.20–2.21)	0.49		
*Vaginal trichomoniasis*	0.68 (0.09–5.18)	0.70		
HSV-2 infection	2.39 (0.32–17.78)	0.39		

a Baseline characteristics.

b Final multivariable model: included all variables with adjusted odds ratio displayed.

#### Regular clients

The adjusted odds of having more than one regular client was significantly reduced during follow-up (aOR=0.42; 95% CI, 0.28–0.63). In parallel, the odds of consistent condom use with regular clients increased with time (aOR=2.18; 95% CI, 1.26–3.76, Table 4).

**Table 4:** Predictors of consistent condom use with regular clients during follow-up

	Univariable model		Final multivariable model^b^	
		
Characteristics	Odds ratio (95% CI)	*p*	Adjusted odds (95% CI)	*p*
Time (3 months)	2.21 (1.36–3.60)	0.001	2.18 (1.26–3.76)	0.005
Age (years)^a^	1.14 (0.71–1.83)	0.57		
Married or cohabiting	0.47 (0.27–0.82)	0.007	0.52 (0.29–0.96)	0.03
Professional sex workers	0.47 (0.26–0.81)	0.007	0.56 (0.31–1.01)	0.05
Education^a^		0.66		
None	1			
1–6 years (primary school)	0.81 (0.45–1.46)			
≥7 years (≥ secondary school)	1.01 (0.53–1.94)			
Age of sex debut^a^	1.15 (1.04–1.27)	0.007		
Duration of sex work (years)^a^	0.97 (0.63–1.52)	0.90		
Number of clients (previous week)	0.98 (0.93–1.03)	0.45		
Number of regular clients (last month)	0.69 (0.57–0.83)	<0.001	0.71 (0.58–0.87)	<0.001
Number of regular partners (last month)	1.94 (1.28–2.95)	0.002	1.97 (1.25–3.09)	0.003
Number of previous pregnancies^a^	0.91 (0.72–1.16)	0.44		
Hormonal contraception	1.44 (0.86–2.41)	0.16		
Previous HIV testing^a^	0.84 (0.51–1.40)	0.50		
Monthly income (€)^a^	0.99 (0.99–1.00)	0.12		
Any alcohol consumption^a^	0.89 (0.56–1.44)	0.64		
Child desire	0.60 (0.35–1.02)	0.05		
Vaginal candidiasis	1.04 (0.47–2.32)	0.91		
Bacterial vaginosis	0.70 (0.32–1.53)	0.36		
*Trichomoniasis vaginalis*	0.69 (0.13–3.53)	0.65		
HSV-2 infection	1.60 (0.33–7.63)	0.55		
Pregnancy during follow-up	0.70 (0.33–1.43)	0.31		

a Baseline characteristics.

b Final multivariable model: included all variables with adjusted odds ratio displayed.

#### Regular partners

After adjustment, the odds of having more than one regular partner at the next follow-up visit was significantly reduced during follow-up (aOR=0.43; 95% CI, 0.29–0.66), without improvement of consistent condom use (aOR=0.71; 95% CI, 0.58–0.96).

## Discussion

Our findings suggest that our model of intervention, integrating prevention and care in the same setting with a strong involvement of the community, had a significant impact on the HIV incidence of young FSW in the capital city of Burkina Faso. Even though this impact was not statistically significant in the extreme and most detrimental scenario, the observed null HIV infection contrasted with the expected number of HIV infections.

The good quality of the data collected in the general population and among clients of FSW at the same time allowed a robust estimation of the HIV incidence expected in the study population in the absence of any targeted intervention.

The “null” HIV incidence during the intervention could result from a low HIV exposure among the study population, particularly among part-time FSW. However, our group and others have reported that such women, barmaids for example, had a risk of HIV infection similar to that of professionals [36]. At screening, the HIV prevalence among young sex workers (7.8%) was about 20 times higher than among females of the same age group in the general population of Ouagadougou (0.4%) [35]. Similarly, their HSV-2 prevalence was also much higher [37], highlighting their risky sexual behaviours. Therefore, the null HIV incidence is likely due to our intervention and not to a selection bias of women with low exposure.

This impact on HIV incidence was likely driven by a reduction in most risky sexual behaviours during the intervention period. The number of sexual partners decreased over time with a concomitant increase in condom use with casual and regular clients during the intervention. However, the intervention could not markedly increase consistent condom use with regular partners. Regular partners and regular clients include different types of men, from “boyfriends” and sex work venues managers to “protectors” who prevent the women from being assaulted at night [2, 38]. The HIV risk is probably high among these men who, in the local context, have many sex worker girlfriends with whom condom use is seldom used. Despite specific sensitization modules on this topic, young FSW are unable to improve their condom negotiation with these partners, most likely because of their vulnerability and willingness to get married. Similarly, the independent negative association between previous pregnancies and consistent condom use with casual clients is probably explained by a reduced condom negotiation power of FSW having dependent children.

Our model of intervention combines prevention and care activities within the same setting, with peers playing a pivotal role in service delivery. A recent review was not able to identify any similar study having reported a facilitated access to ART for FSW in Africa [39]. We strongly believe that the continuum of care proposed in our intervention was crucial to get a high adherence level of FSW to the intervention, as highlighted by the high rate of follow-up for this stigmatized and hard to reach population. Women can trust a whole team of peers and healthcare workers working together, who care for them when necessary (even for routine medical care) rather than referring them to “regular” HIV outpatients’ clinics and health services where they are often stigmatized [11, 40]. In addition, the peer organization provided some support for non-medical issues which are of crucial importance for FSW, such as children schooling, administrative measures and nutritional assistance. Adapted services to the special needs of each study participant, dedicated to prevention and care in general (not only HIV), in a user-friendly and empathetic setting to build confidence and empowerment of FSW, including their self-esteem, are certainly pivotal in the success of interventions targeting FSW.

Our study had a number of limitations. The community involvement contribution was not included in our impact evaluation [19]. In the absence of a control group, the calculation of the expected incidence without intervention is prone to imprecision and information bias on self-report of sexual behaviours in the general population study [41]. Our sensitivity analyses addressed these points and the true HIV incidence likely lies between the ranges of calculated values. The data used for the calculation were collected at the same time as the cohort initiation. The exclusion of women under 18 years and of non-Burkinabe FSW (all full-time FSW) may limit the interpretation of our findings. We cannot exclude a desirability bias in the self-report of sexual behaviours [41]. However, the FSW did not hesitate to report poor condom use with regular partners, even after specific risk-reduction sessions.

We reported previously that the same intervention could achieve high rates of follow-up and virological success among FSW [17], which induced a marked reduction in infectiousness [15]. In this study in Ouagadougou, we showed that this intervention can also markedly have an impact on HIV incidence within a similar but younger population. Our study group reported that this combined intervention is not more expensive than either treating HIV in the general population [42] or funding local NGOs to implement prevention activities. Such a model could also be appropriate for other parts of Africa where sex workers share similar discrimination and limited access to prevention and care [18]. Although the involvement of FSW in HIV dynamics may be lower in other African regions, a recent modelling work suggested that successful interventions targeting FSW could also reduce HIV incidence by half in countries with higher HIV prevalence in the general population [43]. In light of our results, the Burkina Faso health and HIV authorities are scaling up this integrated peer-administered package of interventions at the country level. A proper evaluation of this programme will inform on its cost-effectiveness and relevance when implemented widely in routine.

## Conclusions

An intervention combining peer-based prevention and care within the same setting markedly reduced HIV incidence among FSW in Burkina Faso. This impact was driven by a reduction in the number of regular partners and by increased condom use with clients.

## References

[1] do Espirito Santo ME, Etheredge GD (2003). HIV prevalence and sexual behaviour of male clients of brothels’ prostitutes in Dakar, Senegal. AIDS Care.

[2] Laurent C, Seck K, Coumba N, Kane T, Samb N, Wade A (2003). Prevalence of HIV and other sexually transmitted infections, and risk behaviours in unregistered sex workers in Dakar, Senegal. AIDS.

[3] Alary M, Lowndes CM (2004). The central role of clients of female sex workers in the dynamics of heterosexual HIV transmission in sub-Saharan Africa. AIDS.

[4] Gomes do Espirito Santo ME, Etheredge GD (2005). Male clients of brothel prostitutes as a bridge for HIV infection between high risk and low risk groups of women in Senegal. Sex Transm Infect.

[5] UNAIDS (2010). Global Report UNAIDS report on the Global AIDS epidemic 2010 [Internet].

[6] UNAIDS (2013). Global Report UNAIDS report on the Global AIDS epidemic 2013 [Internet].

[7] Conseil National de Lutte contre le Sida et les Infections Sexuellement Transmissibles (2011). Enquête de surveillance comportementale auprès des groupes à haut risque: Travailleuses du Sexe (TS) et leurs clients au Burkina Faso.

[8] Nagot N, Ouedraogo A, Ouangre A, Cartoux M, Defer MC, Meda N (2005). Is sexually transmitted infection management among sex workers still able to mitigate the spread of HIV infection in West Africa?. J Acquir Immune Defic Syndr.

[9] Nagot N, Ouangre A, Ouedraogo A, Cartoux M, Huygens P, Defer MC (2002). Spectrum of commercial sex activity in Burkina Faso: classification model and risk of exposure to HIV. J Acquir Immune Defic Syndr.

[10] Berthe A, Traore I, Salouka S, Sanou L, Rouamba J, Ouedraogo D (2009). Analyse de la situation de la prostitution et de la réponse au VIH dans le secteur du commerce sexuel á Ouagadougou (Burkina Faso).

[11] Mtetwa S, Busza J, Chidiya S, Mungofa S, Cowan F (2013). “You are wasting our drugs”: health service barriers to HIV treatment for sex workers in Zimbabwe. BMC Public Health.

[12] Alary M, Lowndes CM, Van de Perre P, Behanzin L, Batona G, Guedou FA (2013). Scale-up of combination prevention and antiretroviral therapy for female sex workers in West Africa: time for action. AIDS.

[13] Hawken MP, Melis RD, Ngombo DT, Mandaliya K, Ng'ang'a LW, Price J (2002). Part time female sex workers in a suburban community in Kenya: a vulnerable hidden population. Sex Transm Infect.

[14] Tanser F, Barnighausen T, Grapsa E, Zaidi J, Newell ML (2013). High coverage of ART associated with decline in risk of HIV acquisition in rural KwaZulu-Natal, South Africa. Science.

[15] Nagot N, Ouedraogo A, Weiss HA, Konate I, Sanon A, Defer MC (2008). Longitudinal effect following initiation of highly active antiretroviral therapy on plasma and cervico-vaginal HIV-1 RNA among women in Burkina Faso. Sex Transm Infect.

[16] Cohen MS, Chen YQ, McCauley M, Gamble T, Hosseinipour MC, Kumarasamy N (2011). Prevention of HIV-1 infection with early antiretroviral therapy. N Engl J Med.

[17] Huet C, Ouedraogo A, Konate I, Traore I, Rouet F, Kabore A (2011). Long-term virological, immunological and mortality outcomes in a cohort of HIV-infected female sex workers treated with highly active antiretroviral therapy in Africa. BMC Public Health.

[18] Konate I, Traore L, Ouedraogo A, Sanon A, Diallo R, Ouedraogo JL (2011). Linking HIV prevention and care for community interventions among high-risk women in Burkina Faso – the ARNS 1222 “Yerelon” cohort. J Acquir Immune Defic Syndr.

[19] Laga M, Vuylsteke B (2011). Evaluating AVAHAN's design, implementation and impact: lessons learned for the HIV prevention community. BMC Public Health.

[20] WHO (2012). Prevention and treatment of HIV and other sexually transmitted infections for sex workers in low- and middle-income countries recommendations for a public health approach [Internet].

[21] WHO (2014). Consolidated guidelines on HIV prevention, diagnosis, treatment and care for key populations [Internet].

[22] Boily MC, Pickles M, Lowndes CM, Ramesh BM, Washington R, Moses S (2013). Positive impact of a large-scale HIV prevention programme among female sex workers and clients in South India. AIDS.

[23] Pickles M, Boily MC, Vickerman P, Lowndes CM, Moses S, Blanchard JF (2013). Assessment of the population-level effectiveness of the Avahan HIV-prevention programme in South India: a preplanned, causal-pathway-based modelling analysis. Lancet Glob Health.

[24] Pickles M, Foss AM, Vickerman P, Deering K, Verma S, Demers E (2010). Interim modelling analysis to validate reported increases in condom use and assess HIV infections averted among female sex workers and clients in southern India following a targeted HIV prevention programme. Sex Transm Infect.

[25] Williams JR, Alary M, Lowndes CM, Behanzin L, Labbe AC, Anagonou S (2014). Positive impact of increases in condom use among female sex workers and clients in a medium HIV prevalence epidemic: modelling results from Project SIDA1/2/3 in Cotonou, Benin. PLoS One.

[26] Fox J, White PJ, Weber J, Garnett GP, Ward H, Fidler S (2011). Quantifying sexual exposure to HIV within an HIV-serodiscordant relationship: development of an algorithm. AIDS.

[27] UNAIDS (2010). New HIV infections by mode of transmission in West Africa: a multi-country analysis [Internet].

[28] Meda N, Gautier-Charpentier L, Soudre RB, Dahourou H, Ouedraogo-Traore R, Ouangre A (1999). Serological diagnosis of human immuno-deficiency virus in Burkina Faso: reliable, practical strategies using less expensive commercial test kits. Bull World Health Organ.

[29] Nagot N, Ouedraogo A, Defer MC, Vallo R, Mayaud P, Van de Perre P (2007). Association between bacterial vaginosis and Herpes simplex virus type-2 infection: implications for HIV acquisition studies. Sex Transm Infect.

[30] Boily MC, Baggaley RF, Wang L, Masse B, White RG, Hayes RJ (2009). Heterosexual risk of HIV-1 infection per sexual act: systematic review and meta-analysis of observational studies. Lancet Infect Dis.

[31] UNAIDS (2008). A framework for monitoring and evaluating HIV prevention programmes for most-at-risk populations.

[32] Ghys PD, Diallo MO, Ettiegne-Traore V, Kale K, Tawil O, Carael M (2002). Increase in condom use and decline in HIV and sexually transmitted diseases among female sex workers in Abidjan, Cote d'Ivoire, 1991–1998. AIDS.

[33] Lalonde TL, Nguyen AQ, Yin J, Irimata K, Wilson JR (2013). Modeling correlated binary outcomes with time-dependent covariates. J Data Sci.

[34] Bjork J, Albin M, Grahn P, Jacobsson H, Ardo J, Wadbro J (2008). Recreational values of the natural environment in relation to neighbourhood satisfaction, physical activity, obesity and wellbeing. J Epidemiol Community Health.

[35] Institut National de La Statistique et du Démographie (INSD) Ministère de l'Economie et des Finances (2012). Enqête Demographique et de Santé et à Indicateurs Multiples (EDSBF-MICS IV) 2010.

[36] Chohan V, Baeten JM, Benki S, Graham SM, Lavreys L, Mandaliya K (2009). A prospective study of risk factors for herpes simplex virus type 2 acquisition among high-risk HIV-1 seronegative women in Kenya. Sex Transm Infect.

[37] Kirakoya-Samadoulougou F, Nagot N, Defer MC, Yaro S, Fao P, Ilboudo F (2011). Epidemiology of herpes simplex virus type 2 infection in rural and urban Burkina Faso. Sex Transm Dis.

[38] Wang C, Hawes SE, Gaye A, Sow PS, Ndoye I, Manhart LE (2007). HIV prevalence, previous HIV testing, and condom use with clients and regular partners among Senegalese commercial sex workers. Sex Transm Infect.

[39] Chersich MF, Luchters S, Ntaganira I, Gerbase A, Lo YR, Scorgie F (2013). Priority interventions to reduce HIV transmission in sex work settings in sub-Saharan Africa and delivery of these services. J Int AIDS Soc.

[40] Conseil National de Lutte contre le Sida et les Infections Sexuellement Transmissibles (2010). Cadre Stratégique de Lutte Contre le VIH, le sida et les infections sexuellement transmissibles (CSLS) 2011–2015.

[41] Aho J, Koushik A, Diakite SL, Loua KM, Nguyen VK, Rashed S (2010). Biological validation of self-reported condom use among sex workers in Guinea. AIDS Behav.

[42] Cianci F, Sweeney S, Konate I, Nagot N, Low A, Mayaud P (2014). The cost of providing combined prevention and treatment services, including ART, to female sex workers in Burkina Faso. PLoS One.

[43] Steen R, Hontelez JA, Veraart A, White RG, de Vlas SJ (2014). Looking upstream to prevent HIV transmission: can interventions with sex workers alter the course of HIV epidemics in Africa as they did in Asia?. AIDS.

